# A model for rigorously applying the Exploration, Preparation, Implementation, Sustainment (EPIS) framework in the design and measurement of a large scale collaborative multi-site study

**DOI:** 10.1186/s40352-018-0068-3

**Published:** 2018-04-13

**Authors:** Jennifer E. Becan, John P. Bartkowski, Danica K. Knight, Tisha R. A. Wiley, Ralph DiClemente, Lori Ducharme, Wayne N. Welsh, Diana Bowser, Kathryn McCollister, Matthew Hiller, Anne C. Spaulding, Patrick M. Flynn, Andrea Swartzendruber, Megan F. Dickson, Jacqueline Horan Fisher, Gregory A. Aarons

**Affiliations:** 1 Institute of Behavioral Research, Texas Christian University, Box 298740, Fort Worth, TX 76129 USA; 2 Department of Sociology, University of Texas at San Antonio, San Antonio, TX USA; 3 National Institute on Drug Abuse, Bethesda, MD USA; 4 Rollins School of Public Health, Emory University, Atlanta, GA USA; 5 National Institute on Alcohol Abuse and Alcoholism, Bethesda, MD USA; 6 Department of Criminal Justice, Temple University, Philadelphia, PA USA; 7 Heller School for Social Policy and Management, Brandeis University, Waltham, MA USA; 8 Health Economics Research Group, University of Miami, Miami, FL USA; 9 College of Public Health, Epidemiology and Biostatistics, University of Georgia, Athens, GA USA; 10 Behavioral Science, University of Kentucky, Lexington, KY USA; 11 The National Center on Addiction and Substance Abuse, New York, NY USA; 12 Department of Psychiatry, and Child and Adolescent Services Research Center, University of California, San Diego, CA USA

**Keywords:** Conceptual frameworks, Exploration, Preparation, Implementation, Sustainment, Juvenile justice, Substance use, Data-driven decision making, Facilitation

## Abstract

**Background:**

This paper describes the means by which a United States National Institute on Drug Abuse (NIDA)-funded cooperative, Juvenile Justice-Translational Research on Interventions for Adolescents in the Legal System (JJ-TRIALS), utilized an established implementation science framework in conducting a multi-site, multi-research center implementation intervention initiative. The initiative aimed to bolster the ability of juvenile justice agencies to address unmet client needs related to substance use while enhancing inter-organizational relationships between juvenile justice and local behavioral health partners.

**Methods:**

The EPIS (Exploration, Preparation, Implementation, Sustainment) framework was selected and utilized as the guiding model from inception through project completion; including the mapping of implementation strategies to EPIS stages, articulation of research questions, and selection, content, and timing of measurement protocols. Among other key developments, the project led to a reconceptualization of its governing implementation science framework into cyclical form as the EPIS Wheel. The EPIS Wheel is more consistent with rapid-cycle testing principles and permits researchers to track both progressive and recursive movement through EPIS. Moreover, because this randomized controlled trial was predicated on a bundled strategy method, JJ-TRIALS was designed to rigorously test progress through the EPIS stages as promoted by facilitation of data-driven decision making principles. The project extended EPIS by (1) elucidating the role and nature of recursive activity in promoting change (yielding the circular EPIS Wheel), (2) by expanding the applicability of the EPIS framework beyond a single evidence-based practice (EBP) to address varying process improvement efforts (representing varying EBPs), and (3) by disentangling outcome measures of progression through EPIS stages from the a priori established study timeline.

**Discussion:**

The utilization of EPIS in JJ-TRIALS provides a model for practical and applied use of implementation frameworks in real-world settings that span outer service system and inner organizational contexts in improving care for vulnerable populations.

**Trial registration:**

NCT02672150. Retrospectively registered on 22 January 2016.

## Background

Regardless of service sector or clientele, any initiative aimed at changing public sector systems of care requires careful consideration of potentially challenging factors that commonly impact implementation efforts. Common challenges include structural and cultural impediments to change within organizations, personnel aversion to the adoption of new practices, inadequate information systems, and lack of coordination among agencies within an existing system. The field of implementation science has emerged so that such factors can be identified and addressed in a systematic fashion and, where possible, successfully overcome. Moreover, implementation science permits researchers examining change within complex systems to make critical study design decisions informed by a guiding conceptual framework.

This paper describes how a particular implementation science model developed to facilitate implementation and sustainment in public sectors, *Exploration, Preparation, Implementation, and Sustainment* (EPIS) (Aarons, Hurlburt, & Horwitz, 2011), and was applied within a multi-site, multi-research center initiative. This National Institute on Drug Abuse (NIDA)-funded initiative, Juvenile Justice-Translational Research on Interventions for Adolescents in the Legal System (JJ-TRIALS), used an implementation science approach from its inception with the goal of improving substance use treatment outcomes for justice-involved youth on community supervision (Knight et al., 2016). To foster data-driven decision making principles and interorganizational collaboration using EPIS, this two-arm implementation intervention was completed through the baseline period in spring-2016, through the experiment period in mid-2017, with the post-experiment period concluding fall-2017. We describe the methodology and application of the EPIS framework to the field of juvenile justice and offer a unique example in the application and tailoring of implementation frameworks to a new more complex multisystem administrative domain outside of health, child welfare, or mental health services. This paper aims to illustrate how a guiding framework was used in the development and execution of an implementation intervention project designed to improve service delivery for justice-involved youth who receive services within complex, multi-agency systems.

### Public health issue

The link between criminal activity, high-risk behavior, and substance use is well documented (Belenko & Logan, 2003; Chassin, 2008; Harzke et al., 2012). Approximately 75% of juvenile offenders report a history of drug or alcohol use (Belenko & Logan, 2003), with more than a third meeting criteria for a substance use disorder (Wasserman, McReynolds, Schwalbe, Keating, & Jones, 2010). Despite the high prevalence of substance use disorders among juvenile offenders, many do not receive evidence-based substance use screening, assessment, and treatment (Belenko & Dembo, 2003; Knudsen, 2009). Moreover, untreated juvenile offenders are at a higher risk of recidivating (Hoeve, McReynolds, & Wasserman, 2014; Young, Dembo, & Henderson, 2007), and are likely to exhibit mental health adversities (Chassin, 2008). There are numerous reasons that youth on community supervision do not receive needed services, but evidence suggests that a breakdown in transitioning youth across service systems is a persistent source of unmet client need (Spencer & Jones-Walker, 2004; Staples-Horne, Duffy, & Rorie, 2007).

A comprehensive array of services (ranging from screening and assessment to intensive treatment options) can be provided within a single agency such as a probation department or drug court. However, this approach is rare. Often, youths who could benefit from receiving several types of services must interact with multiple service agencies that operate independent of the juvenile justice (JJ) entity. For instance, youth commonly interact with probation officers and clinical staff who work within independently functioning organizations such as probation departments or behavioral health (BH) agencies. This model represents a possible gap in system-organization relationships and organizational networks. Consequently, services are fragmented, and youth may “fall through the cracks” when transitioning between different service systems and provider organizations.

Distinctive characteristics among these agencies may also obstruct efforts to improve service coordination at multiple levels (Flynn, Knight, Godley, & Knudsen, 2012; Shortell, 2004). Staff who supervise youth may bring personal biases or perspectives (Aarons, 2004) and workplace influences (Becan, Knight, & Flynn, 2012) into their organizational roles, and these can undermine effective service delivery (Williams & Glisson, 2014). Further, organizations may have unique cultures that can impede service quality and prospects for change (Glisson & Hemmelgarn, 1998). Interagency relationships can be influenced by historical antagonisms, changing priorities, and tendencies to “silo” service delivery (Bunger, Collins-Camargo, McBeath, Chuang, Pérez-Jolles, & Wells, 2014). Finally, interagency networks exist within broader regional, state, or local systems while contending with policies that influence public safety regulations, funding mechanisms, progress monitoring, and information sharing.

Many implementation studies focus on the inner context of organizations and staff within them. However, when improvement in client outcomes is contingent on service provision by multiple agencies, strategies should be aimed at all relevant levels across systems and organizations, thereby adding the outer context of the “system” or “community.” The selection of specific agencies and how many levels to target depends in part on the complexity of the service system. For juvenile justice, a key consideration is the degree to which youth must interact with multiple agencies to receive needed services. Likewise, measuring change within each of the targeted levels is paramount.

Conceptual models can be useful for informing the selection of implementation strategies, as well as timing and targets of such strategies (Damschroder, Aron, Keith, Kirsh, Alexander, & Lowery, 2009; Grol, Bosch, Hulscher, Eccles, & Wensing, 2007; The Improved Clinical Effectiveness through Behavioural Research Group (ICEBerg), 2006). Models can also help inform the selection of measures to test strategy effectiveness at multiple outcome levels (Proctor, Landsverk, Aarons, Chambers, Glisson, & Mittman, 2009), including implementation (e.g., practice adoption), service delivery (e.g., effectiveness), and client impacts (e.g., satisfaction). Below we describe how the EPIS framework was utilized throughout JJ-TRIALS to optimize meeting these goals.

## Methods

The JJ-TRIALS Project (Knight et al., 2016) is a multisite cluster randomized trial that aimed to compare the effectiveness of two implementation strategy bundles, namely, site randomization to core or enhanced conditions. Agencies assigned to the enhanced condition received additional strategies, most notably researcher-based facilitation, hypothesized to improve the delivery of substance use services for juvenile offenders on community supervision. Each of the 36 sites (located in 7 states) included a community-based JJ agency and a partnering BH agency. Sites received identical strategies during the baseline period, additional strategies (namely, facilitation) for enhanced sites during the experiment period, and independent continuation of strategies during the post-experiment period. In what follows, we describe why EPIS was selected and illustrate how the model served to guide (a) the overarching study design, (b) the mapping of implementation strategies across the study periods to EPIS stages, (c) the articulation of research questions, and (d) the selection and timing of measurement protocols.

### Selection of an implementation science framework

The JJ-TRIALS project offered a unique opportunity to study the application of implementation science using a specified conceptual model on the process of translating research into practice for the JJ field. The structure of the JJ-TRIALS cooperative was itself an important factor in the ultimate selection of a conceptual model. The cooperative consisted of 6 competitively selected research centers (RCs), each of which recruited one or more state or regional JJ agency leaders to participate as full partners in the planning, execution, and evaluation of the studies (Leukefeld et al., 2017). The JJ partners played an integral role in the cooperative by articulating complex aspects of the JJ system within their states and regions, describing the role of partnerships between JJ and BH agencies, shaping the selection and content of the implementation strategies to be tested, securing buy-in among participating agencies, reviewing measurement and design plans for feasibility and acceptability among partnering agencies, and reminding the local JJ and BH agencies about the true purpose and intent of JJ-TRIALS: to help communities achieve their goal of more effectively meeting the needs of juveniles with substance use problems.

Together with the JJ partners, the lead investigators from each JJ-TRIALS RC and NIDA staff met to consider, assess, and select an implementation science model at the outset of the project (August 2013). During this two-day meeting, several frameworks were carefully considered based on a then-recent review by Tabak and colleagues (Tabak, Khoong, Chambers, & Brownson, 2012). The group consensus was to identify a model that: (1) featured clearly delineated stages, (2) recognized the multi-level nature of service delivery systems in which multiple actors/agents are nested within organizations, systems, and broader environmental contexts, and (3) was suitable for use in the primary target organizations (juvenile justice agencies) with minimal burden to agency staff (Flynn et al., 2012).

EPIS was selected as the optimal conceptual model (Nilsen, 2015) for JJ-TRIALS because it allows for examination of a change process at multiple levels, across time, and through successive stages that build deliberately toward implementation, while discerning longer-term impacts in the form of sustainment. EPIS, as utilized here, describes implementation as a process moving through four stages (Aarons et al., 2011): Exploration (identifying practices to be implemented, assessing system, organization, provider, and client-level factors that explain service gaps and potential barriers/facilitators for change); Preparation (redesigning the system to enhance service availability and ensure consistent implementation of proposed changes); Implementation (training, coaching, and active facilitation of evidence-based practices [EBPs] to be adopted); and Sustainment (maintaining the use of the newly installed practices). In each stage, EPIS identifies factors relevant to implementation success in both the inner context (i.e., within the implementing organization itself) and outer context (i.e., external to, but still influential on, the implementing organization) and their interactions (Aarons et al., 2011).

Selection of the EPIS framework was, in part, intended to respond to the needs of the organizational settings (i.e., JJ agencies) in which it was to be applied. Because agency staff would themselves serve as the change agents, it was important that the selected model avoid imposing complexity that would overwhelm or burden them. JJ partner perspectives were critical in estimating the degree of burden likely to occur at research sites due to the chosen implementation model, thereby underscoring the value of research/practitioner relationships from the outset. EPIS provides a comprehensive four-stage framework that is readily understood by organizational actors who are asked to draw on their local knowledge of agency-level and system-level dynamics to create sustainable change.

### EPIS as a guide for overarching study design

EPIS was selected as a guiding framework prior to the design of the JJ-TRIALS study, and therefore drove the selection, design, and timing of every essential component of the study (see detailed intervention protocol) (Knight et al., 2016) and was structured to test underlying assumptions of EPIS. Several strategies were carefully considered in development of the overall study design; strategies were identified by conducting an extensive review of related research including a then recent review (Powell et al., 2012) and completing a series of structured discussions on investigator experiences with identified strategies. Table 1 offers a condensed Implementation Strategies Matrix that summarizes the selected implementation strategies (Proctor, Powell, & McMillen, 2013) and how they were mapped to the study time period and EPIS stage. This careful consideration served to set up the JJ-TRIALS study to rigorously test the EPIS framework in a way that is rare for conceptual frameworks in the field. Below are several examples of how EPIS further guided the development of the JJ-TRIALS study design.

**Table 1:** JJ-TRIALS Implementation Strategies Matrix

Strategy Applied for Both Conditions	Study Period	EPIS STAGES	Strategy Targets
1. Formation of interagency collaboratives and coalitions	Baseline	ALL EPIS STAGES	Interagency workgroups comprised of both JJ and BH representatives including agency leaders (e.g., administrators) and line staff (e.g., probation officers, counselors, data personnel)
2. Local Needs Assessment and Site Feedback Report	Baseline	Exploration	Researchers and interagency workgroups
3. Learning collaborative	ALL STUDY PERIODS	All EPIS STAGES	Researchers, state-level JJ administrators (“JJ partners”), and interagency workgroups
4. Strategic planning	Baseline	Preparation	Interagency workgroups
5. Data-driven decision making (DDDM)	ALL STUDY PERIODS	All EPIS STAGES	Interagency workgroups
6. Plan-Do-Study-Act (PDSA)	Experiment, Post-Experiment	Implementation, Sustainment	Interagency workgroups
Strategy Applied for Enhanced Condition	Study Period	EPIS STAGES	Strategy Targets
7. Local change team	Experiment, Post-Experiment	Implementation, Sustainment	Local change teams comprised of both JJ and BH representatives (Enhanced intervention only)
8. Implementation facilitator	Experiment	Implementation	External Research-Based Facilitators and local change teams
9. Ongoing training and support	Experiment	Implementation	External Research-Based Facilitators and local change teams
10. Local champion and leadership training	Experiment	Implementation	External Research-Based Facilitators and site identified JJ or BH local champion(s) of the local change team

This matrix identifies ten evidence-based implementation intervention strategies that were utilized in the JJ-TRIALS project. This matrix includes (1) the implementation strategy name, (2) primary study period(s), EPIS stage(s), and (3) strategy action targets

#### Linear and dynamic applications

**Fig. 1:**
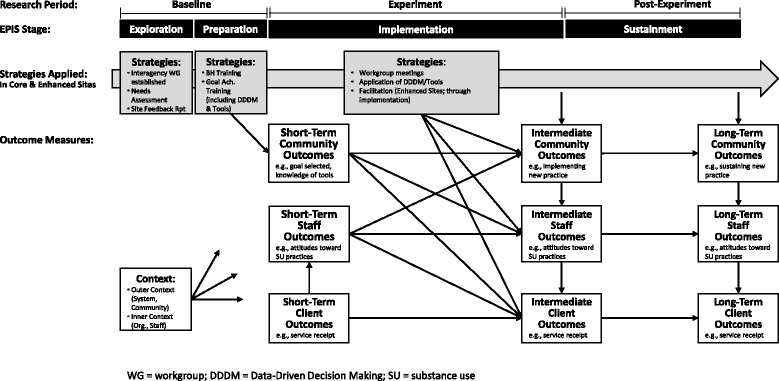
JJ-TRIALS linear application of EPIS

EPIS was developed with a focus on implementation in public sector service systems, although it has since been used in medical and other settings. EPIS is based on a comprehensive review of relevant literature, and builds on several of its predecessors (Damschroder et al., 2009; Proctor et al., 2011; Proctor et al., 2009; Simpson & Flynn, 2007). EPIS integrates various elements into a model that incorporates multi-level system approaches to change over time, across its four stages. Following the EPIS model, it was expected that progress toward system improvement would entail agencies’ investment of time and resources during Exploration, Preparation, Implementation, and Sustainment stages. The design therefore delivers pertinent implementation strategies that correspond with each EPIS stage and measure key variables (e.g., outer and inner context indicators; community, staff, and client outcomes) at the beginning of Exploration and at the end of each subsequent stage. Figure 1 depicts strategies (grey boxes) that correspond with EPIS stages and timing/general content of assessment measures (white boxes; corresponding to community, staff, and client levels). This linear application of EPIS enables examination of (a) the effectiveness of strategies delivered during specific stages, (b) primary hypotheses indicating that the strategy bundle would result in differential outcomes after the experiment and post-experiment periods, and (c) potential effects of outer and inner context factors on implementation, process, and service outcomes.

**Fig. 2:**
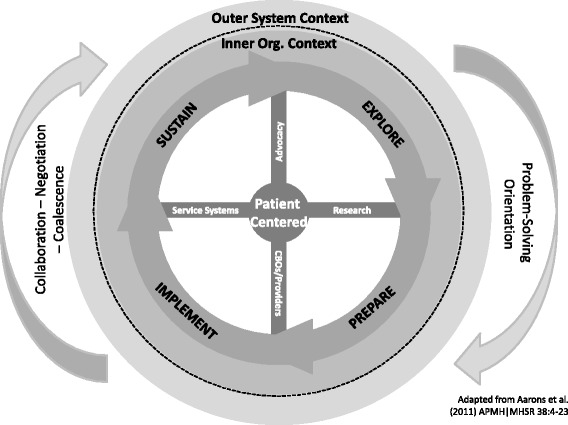
JJ-TRIALS dynamic application of EPIS

As part of an extensive in-person discussion between the JJ partners, RC investigators, NIDA staff, and the EPIS lead author (Gregory Aarons), dynamic processes were overlaid on the linear application. This discussion focused on the degree of flexibility in the model and how it could best reflect the complex and dynamic nature of the participating JJ systems. At the suggestion of the engaged JJ partners, EPIS was adapted from its original linear format with a clearly delineated start point (Exploration) and end point (Sustainment) to a circular wheel-like model (see Fig. 2). Thus, while the clockwise arrows are intended to represent an ideal progression—namely, movement through the stages that eventually gives way to Implementation and ultimately Sustainment—organizational change often occurs in a manner that is not uniformly progressive and may require ongoing adaptation and assessment of adaptation outcomes.

The EPIS Wheel therefore depicts the circular nature of rapid-cycle testing (using iterative cycles to test quality improvement changes on a smaller scale), wherein persistent enhancements and refinements in organizational functioning are encouraged. Furthermore, several stakeholder spokes were added to highlight and represent diverse interests relevant to the JJ context throughout the process of phased change already included in EPIS: (1) advocacy groups, (2) researchers, (3) community-based organizations, and (4) service systems. These spokes conceptualize the change process as dynamic in nature, whereby efforts continually unfold as aspects of the inner and outer contexts evolve and stakeholders at various levels become involved in implementation of goals and objectives. Changes in the system, the organization, or the specific needs of youth can impact the feasibility of conceived implementation efforts. For example, turnover among staff who are responsible for change can slow progress, and new leadership can shift priorities or redefine objectives so that resources for implementation are no longer available. When these developments occur, the stakeholders must naturally revisit previous stages, revise plans or even explore new options.

The outer rim of the EPIS Wheel captures this dynamic movement in either a progressive (forward/linear) direction or a recursive (backward/cyclical) direction as “problem-solving orientation” and “collaboration-negotiation-coalescence” that may characterize the nature of successful community-academic collaborations (Aarons et al., 2014). Recursive activity is inherent to multi-agency collaborations and feedback loops, and as such it is not only planned in JJ-TRIALS as part of the implementation strategy bundle (e.g., use of rapid-cycle testing), but can apply to the entire change process. Because recursive movement can solidify foundational change elements that eventually spur significant quality improvement, recursivity was expected and examined for its effects on change. As described in more detail later, one central methodological innovation of JJ-TRIALS lies in the development of measurement strategies to capture this recursive movement.

### Mapping of implementation intervention strategies to EPIS stages

One challenge in utilizing EPIS as a guiding conceptual model entailed determining how to map the EPIS stages to study activities (Baseline, Experiment, Post-Experiment; see Fig. 1), while allowing for expected diversity across the sites on a number of dimensions (e.g., selection of process improvement goals, progression through EPIS stages). Because EPIS asserts that the effectiveness of implementation efforts are in part dependent on activities that occur during Exploration and Preparation, the Cooperative decided that all 36 JJ-TRIALS sites should receive identical support strategies during the Baseline period (targeting Exploration and Preparation activities). An identical set of Baseline activities allowed a novel opportunity to compare the effectiveness of two implementation interventions (delivered during the Experiment period) on successful movement through the Implementation stage as either bolstered by facilitation (enhanced condition) or as acting independently without external facilitation (core condition). Keeping with the EPIS framework, the study timeline incorporated a Post-Experiment period during which sites were monitored in their independent efforts to continue new practices (Sustainment stage). Disentangling the study methodology of a priori study period, in which implementation strategies would be applied, from the overarching conceptual basis of this study (site progression through EPIS) was an inherent challenge and contribution of the JJ-TRIALS project. Below, we describe the integration of the conceptual framework with the study methodology, including essential study activities and outcomes, as they were cross-mapped onto the EPIS framework.

#### Baseline period: Exploration

The initial implementation intervention activities delivered during the JJ-TRIALS Baseline period corresponded to the Exploration phase of EPIS. Consistent with EPIS’s emphasis on inter-organizational networks (Aarons et al., 2011), JJ agencies were asked to identify one or more BH agencies in their local community that (a) provided substance use services (e.g., comprehensive assessment, treatment) and (b) would be willing to collaborate with JJ to increase service access and receipt. Following identification of a local BH partner, an interagency workgroup was established, comprised of leadership and line level stakeholders from both JJ and BH agencies. Early in the Baseline period, RCs obtained youth service-level data from agencies, workgroups participated in an RC-led comprehensive needs assessment of existing services, and staff at participating agencies were offered training on the “Behavioral Health Services Cascade” (herein referred to as “the Cascade”) (Belenko et al., 2017)—a framework for provision of best practices for addressing substance use that emphasizes an optimal service continuum from initial screening, comprehensive assessment, and referral to appropriate level of care. Data from the needs assessment, plus information on receipt of services along the Cascade from JJ and BH agency youth record systems were used by RCs to generate a site-specific, written feedback report. The report served as both a snapshot of baseline service receipt, identifying areas of greatest unmet substance abuse treatment needs, and as a measurement feedback tool (Douglas, Button, & Casey, 2016) for demonstrating data-driven decision making (DDDM) in selection of a process improvement goal.

#### Baseline period: Preparation

After completing the activities described above, participating sites identified relevant goals to pursue. Interagency workgroups met to examine their own service systems and receive training on strategies for process improvement efforts (e.g., goal selection, DDDM). The Goal Achievement Training occurred over a two-day onsite meeting during which RCs provided support to agencies for (1) identifying realistic goals and actionable steps for implementing new service practices along the Cascade as informed by the site feedback report, (2) using DDDM to inform progress toward site goals and steps, and (3) sustaining changes in delivery of substance use services. Subsequent to goal selection, sites were asked to develop an action plan, which specified the concrete, measurable steps required to attain their goal. The conclusion of training signaled the close of the Baseline period, and thus the end of the identical support strategies across the 36 sites. The study design specified that successful selection of a goal and initiation of work on an action plan during training would signal entry into the Implementation stage of EPIS, whereas sites that did not select a goal would remain in the Exploration stage as the Experiment period began. Ultimately, all sites were successful in goal selection and initiated work toward defining steps during training. Because the study periods are conceptualized as separate from the EPIS stages, this project tracks how various intervention components affect movement through EPIS.

#### Experiment period: Implementation

At the close of the Baseline period, all sites were asked to work toward their site identified goals (implementation of new service practices) and to utilize JJ-TRIALS tools and resources for the following 12-month Experiment period. For example, sites were encouraged to apply DDDM techniques including rapid-cycle testing (Deming, 2000) whereby proposed changes are carefully pilot-tested in a bounded context and, as needed, modified before being brought more fully to scale. The DDDM techniques included worksheets to help sites identify what they are trying to accomplish, how they will know that change is an improvement, what changes can be made to result in improvement, what went well and what problems occurred once tested, what the data reflect about implementation, and ultimately if the change resulted in improvement (Horan Fisher, Becan, Harris, Nager, Baird-Thomas, Hogue et al., in press). Therefore, although sites were technically working in the Implementation stage, by design, rapid-cycle testing allowed recursive movement such that sites could cycle to the earlier Exploration and Preparation stages as refinements to agency plans and procedures were made. For instance, through early use of the JJ-TRIALS DDDM tools, a site discovered that their initial plan to increase referral to the appropriate level of care required adaptation to include a more sensitive and evidence-based assessment process. This discovery necessitated site recursive activity to the earlier stage of Exploration to identify an assessment tool and Preparation plans for adoption.

To test the primary hypotheses (comparing the two implementation intervention strategies), half of the sites (those randomized to the enhanced condition) received access to a researcher-based facilitator during the Experiment period. Facilitators’ roles included cultivating ongoing workgroup discussions, supporting and encouraging the use of data to inform agency decisions and to monitor and inform site goals, providing guidance in progress toward site identified process improvement plans, and promoting sustainment of site changes such as documenting changes in site policy and procedures and developing plans to roll out piloted service revisions site-wide (Knight et al., 2016).

#### Post-experiment period: Sustainment

Access to facilitators for the enhanced sites was removed after the 12-month Experiment period. To potentially enable examination of sustainment, sites’ activities continued to be tracked across the following 6-month Post-Experiment period. During this period, all sites were encouraged to continue working toward their goal (if not yet achieved), work toward sustainment of enacted changes, implement changes agency-wide (i.e., bring changes to scale), and/or identify and work toward other site-identified goals to address unmet service needs among youth on community supervision. Utilization of DDDM tools toward identified goals occurred independent of researcher-based facilitation in all sites, which allowed natural observation of site continued engagement in process improvement activities.

### EPIS informs articulation of research questions

As with the timing and selection of implementation strategies, the EPIS framework informed the development and articulation of research questions and hypotheses. In addition to primary hypotheses—examining the differential effect of the strategies bundle on sites with (enhanced condition) and without researcher-based facilitation (core condition)—using EPIS as a guiding framework stimulated exploratory research questions regarding movement across and within EPIS stages, as well as how that process impacted implementation outcomes (i.e., rates of service delivery along the Cascade). For instance, exploratory questions inspired by a linear application of EPIS included “Relative to core, does the enhanced condition increase the likelihood that sites will implement their action plans by the end of the Experiment period?” and “Is implementation of action plans associated with improved outcomes during the Post-Experiment period?” It was hypothesized that without support in the form of facilitation, core sites may get “stuck” at the Preparation stage and not move fully into the Implementation or Sustainment stages. Questions inspired by a dynamic application of EPIS, highlighting the recursive process of change, included “Relative to core, does the enhanced condition improve workgroup perceptions of the value of DDDM?” and “If DDDM is used, does it promote improved outcomes during the Post-Experiment period?” Rapid-cycle testing can be a lengthy and burdensome process, especially if workgroup members become discouraged when progress diminishes during recursive activity. Facilitation may help keep workgroups on target and help maneuver such challenges.

Other questions inspired by dynamic applications included “Are sites that engage in more recursive movement (e.g., multiple revisions to plans) more successful in increasing youth services?” and “Do workgroups that function collaboratively with stakeholders at various levels of the partnering agencies (i.e., BH agency partners, juvenile probation officers) revise plans more substantially and implement more sustainable plans compared to workgroups with representation from a single agency (i.e., mostly JJ staff) or single level (e.g., only leadership)?” Expanding beyond examination of linear movement provided a novel opportunity to test the relationship between recursive activity within or across EPIS stages, engagement in process improvement activities (e.g., use of data to inform decisions, stakeholder efforts), attitudes toward continued use of process improvement methods, and sustainment of new practices.

### Selection and timing of measurement protocols across EPIS stages

A measurement design challenge inherent in working with an implementation study involving varying process improvement goals (e.g., services along the Cascade) and assessment of change across all four EPIS stages involved how to predict and articulate *which* domains/variables are most important to measure at *which* points over time as change efforts unfold. Design and measurement choices clearly needed to be made a priori but, in some cases, modified throughout the project (e.g., monitoring and minimizing burden on agency partners is an important consideration). Prior empirical research can certainly help guide these decisions and assess different tradeoffs to some degree, depending upon the specific process improvement goal and the setting under consideration. Consequently, the central research questions guiding JJ-TRIALS and the project’s data collection procedures were ultimately informed by EPIS.

#### Linear measurement applications

**Fig. 3:**
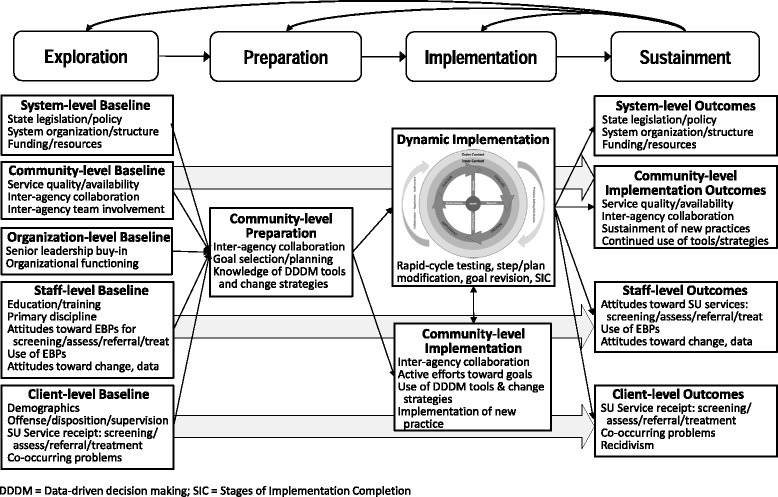
JJ-TRIALS measurement framework of EPIS stages

Linear measurement applications specific to JJ-TRIALS were based on Aarons’ original conceptualization (Aarons et al., 2011) depicted in Fig. 3 and Table 2. To examine hypotheses, JJ-TRIALS used a nested contexts approach featuring methodological processes at five critical levels: (1) system, (2) community, (3) organization, (4) staff, and (5) client. These levels are captured in EPIS as outer context (i.e., system and community) and inner context (i.e., organization, staff, client). Additionally, rather than simply measuring change at two time points (baseline and post-experiment periods), in line with the EPIS staged model, data were collected at regular intervals to examine changes and progression across each stage of the EPIS framework.

**Table 2:** Measurement Matrix: Adaptation of EPIS Framework to JJ-TRIALS

				26-Month Span			
				Baseline		Experiment	Post-Exp.
Domain	Constructs	Instruments	Description	EXPL (mo. 1–5)	PREP (mo. 6–7)	IMPL (mo. 8–19)	SUST (mo. 20–25)
Outer Context (System and Community Levels)	System-level Factors	Agency Survey (JJ/BH)	Sociopolitical (legislation, policies, audits); Funding (sources, insurance, grants)	Month 1			Month 20
	Inter-organizational Networks	Staff Survey (JJ/BH)	Frequency & quality of multi-agency communication	Month 5	Month 9	Month 19	Month 24
	Staff Value and Use of Process Improvement	Staff Survey (JJ/BH)	Staff importance and use of process improvement approaches	Month 5	Month 9	Month 19	Month 24
	Engagement in Process Improvement	Monthly Site Check-in	Workgroup progress toward goals, use of process improvement tools, and staff time invested in process improvement			Months 8–19	Month 20–24
Inner Context	Engagement in Process Improvement	Focus Group	Group interview on progress toward goals			Month 19	Month 24
(Organization and Staff Levels)	Organizational Characteristics	Agency Survey (JJ/BH)	Agency capacity, number of staff	Month 1			Month 20
	Organizational Functioning	Staff Survey (JJ/BH)	Organization climate, leadership	Month 5	Month 9	Month 19	Month 24
	Individual Adopter (staff) Characteristics	Staff Survey (JJ/BH)	Demographics and Change- Oriented Attributes	Month 5	Month 9	Month 19	Month 24
	Staff Value and Use of EBP	Staff Survey (JJ/BH)	Staff importance and use of EBP	Month 5	Month 9	Month 19	Month 24
	Service Quality	Agency Survey (JJ/BH)	Agency Survey on current services	Month 1			Month 20
	Service Quality	Needs Assessment	Group interview on flow of current services	Month 3			Month 20
Client Level Service Receipt	Service Rates	Youth Service Records	Receipt of services (%, timing)	Month 1, 4	Month 7	Month 10, 13, 16, 19	Month 22, 25

Approximate EPIS Stage: *EXPL* Exploration Phase, *PREP* Preparation Phase, *IMPL* Implementation Phase, *SUST* Sustainment Phase

Community measures allowed for examination of implementation outcomes (Proctor et al., 2011), including engagement in process improvement activities such as JJ and BH agency representation at workgroup meetings and site initiated use of DDDM. Organization measures provided markers for implementation outcomes (e.g., perceived EBP appropriateness, adoption/initial uptake, penetration across agency personnel, and sustainment and scaling up of new EBPs). Client-level indicators (e.g., service rates) provided service outcome measures of effectiveness (Proctor et al., 2011). The nested contexts approach to methodological processes provided an opportunity for examining site initiated use of JJ-TRIALS strategies and tools, independent of condition assignment, and examination of the dynamic applications of EPIS, as described below.

#### Dynamic measurement applications

Dynamic applications for measurement are represented by the two boxes below “Implementation” in Fig. 3. The top box depicts the EPIS Wheel and encompasses measures aimed at quantifying recursive activities. The bottom box includes more straightforward measures—use of tools, progression through the Implementation stage, and fidelity to the study protocol.

#### Quantifying recursive activity

Quantifying recursive activity involved documenting activities and coding them to capture elements of the cyclical movement depicted in Fig. 2. Site activities and benchmarks were documented during experiment and post-experiment periods using monthly site check-in calls and site maintained activity logs. During monthly calls, RCs solicited information from sites on changes in staffing, data collection, and client services (including funding, referrals, budget, and case management). Information on inner context factors impacting progress toward process improvement goals, staff time allocated to workgroup meetings and independent JJ-TRIALS project related tasks, and use of DDDM tools were solicited. The site maintained monthly activity logs, as shared with RCs, included more detailed information on changes to the action plan/steps (additions, deletions, revisions) and forward or recursive movement in process improvement activities (completion of steps, returning to completed steps, engagement in rapid-cycle testing). Focus groups with interagency workgroup members were conducted by RC lead investigators at the conclusion of the post-experiment period to document continued engagement in process improvement activities including sustained use of services/practices and site roll-out of goals and action plans developed through JJ-TRIALS involvement. The attempt to measure recursive movement across and within EPIS stages and during experiment and post-experiment periods is novel to this project and will allow examination of the value and effectiveness of implementation intervention strategies including data-driven decision making.

#### Transition markers between EPIS stages

**Fig. 4:**
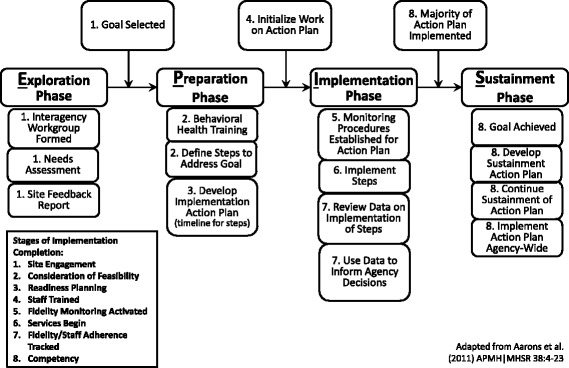
JJ-TRIALS conceptual framework of EPIS stages and transition points

The decision to allow sites to select a goal that corresponded to any point in the Cascade (i.e., improving screening, assessment, referral, or treatment practices) posed significant measurement challenges. This allowance meant that site-selected EBPs would also vary, and represented a departure from studies applying EPIS to the implementation of a single EBP across multiple sites (Aarons et al., 2016). However, there is a precedent for studies examining implementation and sustainment of multiple EBPs as a function of large scale policy initiatives (Lau & Brookman-Frazee, 2016). The JJ-TRIALS lead investigators, in collaboration with originators of the Stages of Implementation Completion (SIC; Lisa Saldana) framework and measure (Saldana, Chamberlain, Bradford, Campbell, & Landsverk, 2014), and the EPIS framework (Gregory Aarons), cross-mapped the SIC activities to specific stages of the EPIS framework and to specify/code activities universal to differing goals. Cross-mapping the SIC to EPIS allowed for a more precise analysis of agency movement across the four EPIS stages through examination of the degree to which activities occurred during each stage. Figure 4 demarcates study-related activities by EPIS stage with numeric indication of SIC stages for each activity. For instance, sustainment is measured in JJ-TRIALS by goal attainment, with an emphasis on completing the majority of steps, rather than commonly used sustainment measures such as training, fidelity, and competence which are typically packaged with a specified EBP (Aarons et al., 2016; Brookman-Frazee et al., 2016; Chamberlain, Hendricks Brown, & Saldana, 2011; Novins, Green, Legha, & Aarons, 2013). While sites prioritized varying service changes along the Cascade (e.g., adoption of a screening tool, assessment tool, or referral process), all sites developed an action plan with goal steps to plan, implement, study, and revise. These action plans, with specified goal steps (e.g., purchase tool, establish implementation procedures, train staff on process, formalize protocol), allowed for a common metric to document (through monthly site check-in calls) where sites were in the process of implementing their goal. This approach also permitted the research team to develop a common framework by which to compare movement through the EPIS and SIC stages across sites. As a concrete example, site A planned to double their current referral rate for youth on community supervision who were in need of services, and site B planned to decrease the time between completion of the screener and referral for those in need of services. Both sites A and B included an action step to train JJ staff on how to implement and code a newly adopted screening tool. Progression toward training completion indicated movement for each site through important stages of the EPIS and SIC models. Coding/analyses of site progress in these universal activities is currently underway.

Beyond the identification of universal activities common across site selected EBPs, it was crucial to measure objective criteria (e.g., site initiated activities) that could then be used to signal transition between EPIS stages. Developing objective markers of the transition between EPIS stages rather than relying on assumptions about how long each stage should last (e.g., conflating study timeline with site progression toward sustainment) was one of the major methodological issues that had to be resolved in applying and rigorously testing the EPIS framework. As indicated in Fig. 4 with boxes and arrows between stages, benchmarks demarking transition points between stages were tied to *site initiated* activities that coincided with planned *study* activities. For instance, selection of a goal by the workgroup was used as an empirical indicator of movement from Exploration to Preparation. While this benchmark was identically supported by RC intervention activities (e.g., training) in all sites; the transition from Implementation to Sustainment was differentially supported by RC intervention activities (e.g., researcher-based facilitation for enhanced sites) based on random assignment to condition. Therefore, this project allowed transition to occur along two dimensions, *study* activities (e.g., the Post-Experiment period began at end of 12 months of facilitation), and *site-initiated* activities (e.g., the Sustainment stage of the EPIS framework began when a site implemented a majority of their action plan). Allowing a second dimension, namely site-initiated activities, to monitor transitions, provided for a flexible application and empirical test of the EPIS framework and natural variation in the speed through which sites addressed action steps and accomplished their goal. It was expected that some sites, especially the enhanced sites, would transition to the Sustainment stage prior to the end of the 12-month facilitation period.

The a priori designations for signaling transitions between EPIS stages were crucial from a project management perspective. The JJ-TRIALS design allowed for cross-site variations in many ways, but it was also structured to ensure that the overall study moved forward regardless of whether sites met their goals. For example, if a site failed to reach their goal by the end of the Experiment period, this outcome was treated as data rather than a justification for extending the amount of time that site was tracked or extending facilitation that was provided to enhanced sites.

### Design limitations and challenges

As is demonstrated above, the JJ-TRIALS Project with 36 sites, situated across seven states and using EPIS as a guiding framework has successfully conducted one of the largest implementation studies to date. A design challenge inherent to multi-site, multi-stage projects targeting strategies at each successive stage, such as in the JJ-TRIALS project, was the balance between intervention and data collection comprehensiveness and experienced participant and research burden. For instance, while measuring recursive movement through the EPIS model is a key contribution of this project, to minimize site burden, limitations were set with regard to frequency of data and level of detail on activities collected. Data were captured on a monthly basis, rather than in a real-time log of activities; possibly resulting in some loss of information, recall bias, and data inconsistencies within and across sites. Along the same lines, to avoid undue burden, this project restricted estimated implementation costs to investment in the intervention being implemented. Other possible implementation costs (availability of staff time, travel time, supplies, and space) associated with activities across each of the EPIS stages, were seen as too laborious to accurately collect for this project. While there were few approaches for examining implementation costs at the inception of JJ-TRIALS (Liu et al., 2009), recent developments may make implementation cost estimation more practical in future studies (Saldana et al., 2014).

An additional challenge requiring careful consideration for the JJ-TRIALS project was the early emphasis on allowing sites to select EBPs that were responsive to context-specific needs. This focus on context-specific needs was critical for understanding adaptations and tailoring analyses for relevance to each local context. The study design, therefore, placed greater emphasis on tracking process outcomes and resulting agency level EBP adoption, rather than an emphasis on monitoring fidelity; adaptation to promote fit (Chambers & Norton, 2016) (e.g., examination of intervention principles rather than “locked down” interventions) (Mohr et al., 2015); and quality of the practice improvement goals (utilized EBPs).

## Discussion

EPIS is a well-established model for understanding, researching, and supporting the implementation of new practices. It was initially designed for implementation science research focused on public sector services including mental health and child welfare, and similarly organized systems such as substance use disorder treatment (Aarons et al., 2011). As demonstrated herein, the EPIS framework is applicable and transferrable to other service delivery systems, including juvenile justice and youth behavioral health (Knight et al., 2016). EPIS was used to guide (1) the overarching JJ-TRIALS study design, (2) the mapping of implementation strategies across the study periods to EPIS stages, (3) the articulation of research questions, and (4) the selection and timing of measurement protocols. JJ-TRIALS also offered three significant augmentations to the EPIS framework. The project extended EPIS by (a) elucidating the role and nature of recursive activity in promoting change (yielding the circular EPIS Wheel), (b) expanding the applicability of the EPIS framework beyond a single EBP to address varying process improvement efforts (representing varying substance use services), and (c) disentangling outcome measures of progression through EPIS stages from the a priori established study timeline. Using a theoretical model to inform every essential element of a study including the elucidation of recursive activity and disentanglement of outcome measures from the study timeline can be considered universally appropriate and valuable whether the study targets implementation of a single practice or multiple/varying practices.

### Implications

The JJ-TRIALS project represents an ambitious attempt to improve services for justice-involved youth in a number of juvenile justice systems across the United States through a structured organizational and system change initiative. JJ-TRIALS also represents an attempt to advance implementation science. The deliberate and comprehensive integration of a conceptual model into every essential aspect of the JJ-TRIALS study design from inception to completion allowed for a salient examination of implementation, service, and client outcomes. Future efforts will empirically examine the EPIS framework through hypotheses on linear and dynamic movement in process improvement planning. Additional efforts are underway to examine similar processes through a JJ-TRIALS pilot study to promote expansion of HIV/STI education and testing services among juvenile justice and partnering public health agencies. It is the hope that this paper will not only offer a valuable example of how to incorporate a conceptual framework into a complex study design, but also as a platform for rigorously testing models such as EPIS so that they can more effectively inform and advance future implementation efforts.

## Abbreviations

BH: Behavioral Health
Cascade: Behavioral Health Services Cascade
DDDM: Data-Driven Decision Making
EBP: Evidence-based practice
EPIS: Exploration, Preparation, Implementation, Sustainment framework
HIV: Human Immunodeficiency Virus
JJ: Juvenile Justice
JJ-TRIALS: Juvenile Justice—Translational Research on Interventions for Adolescents in the Legal System
NIDA: National Institute on Drug Abuse
NIH: National Institutes of Health
RC: Research Center
SIC: Stages of Implementation Completion
STI: Sexually Transmitted Infection
